# Influence of IL-18 and IL-10 Polymorphisms on Tacrolimus Elimination in Chinese Lung Transplant Patients

**DOI:** 10.1155/2017/7834035

**Published:** 2017-01-26

**Authors:** Xiaoqing Zhang, Jiandong Xu, Junwei Fan, Tao Zhang, Yuping Li, Boxiong Xie, Wei Zhang, Shengtao Lin, Ling Ye, Yuan Liu, Gening Jiang

**Affiliations:** ^1^Department of Pharmacy, Shanghai Pulmonary Hospital, Tongji University School of Medicine, Shanghai, China; ^2^Department of Pharmacy, Jiangwan Hospital of Shanghai Hongkou District, Shanghai, China; ^3^Department of General Surgery, Shanghai First People's Hospital, Shanghai Jiaotong University, Shanghai, China; ^4^Department of Thoracic Surgery, Shanghai Pulmonary Hospital, Tongji University School of Medicine, Shanghai, China

## Abstract

*Aims.* The influence of interleukin-10 (IL-10) and interleukin-18 (IL-18) polymorphisms on tacrolimus pharmacokinetics had been described in liver and kidney transplantation. The expression of cytokines varied in different kinds of transplantation. The influence of IL-10 and IL-18 genetic polymorphisms on the pharmacokinetic parameters of tacrolimus remains unclear in lung transplantation.* Methods.* 51 lung transplant patients at Shanghai Pulmonary Hospital were included.* IL-18* polymorphisms (rs5744247 and rs1946518), IL-10 polymorphisms (rs1800896, rs1800872, and rs3021097), and* CYP3A5 *rs776746 were genotyped. Dose-adjusted trough blood concentrations (*C/D* ratio, mg/kg body weight) in lung transplant patients during the first 4 postoperative weeks were calculated.* Results*. IL-18 rs5744247 allele C and rs1946518 allele A were associated with fast tacrolimus metabolism. Combined analysis showed that the numbers of low IL-18 mRNA expression alleles had positive correlation with tacrolimus* C/D* ratios in lung transplant recipients. The influence of IL-18 polymorphisms on tacrolimus* C/D* ratios was observed in CYP3A5 expresser recipients, but not in CYP3A5 nonexpresser recipients. No clinical significance of tacrolimus* C/D* ratios difference of IL-10 polymorphisms was found in our data.* Conclusions. IL-18* polymorphisms may influence tacrolimus elimination in lung transplantation patients.

## 1. Introduction

Lung transplantation is the ultimate treatment option for those with end-stage lung diseases. Unfortunately, the median survival time after lung transplantation is just over 6 years [1–3]. Long-term patient and graft survival are limited by both acute and chronic lung allograft rejection. Current immunosuppression regimen lifelong used in the majority of lung transplant recipients comprised a calcineurin inhibitor (e.g., tacrolimus), an antimetabolite (e.g., mycophenolate mofetil), and corticosteroids [4]. Tacrolimus binding to a cytoplasmic protein, FK506-binding protein 12 (FKBP12), formed a complex. This complex inhibited transcription of cytokines, including interleukin-2, and ultimately blocked T-lymphocyte activation and proliferation. Tacrolimus-based immunosuppressive therapy was characteristic of narrow therapeutic index and broad interindividual variability. Underimmunosuppression resulted in allograft loss while overimmunosuppressive therapy caused infection, lymphoproliferative disease, and multiple drug toxicities (e.g., renal dysfunction, diabetes mellitus, dyslipidemia, obesity, and arterial hypertension) [5, 6]. Therefore, tailoring immunosuppression to an individual patient might improve the outcome of lung transplantation.

Pharmacogenetics, which is the study of genetic differences affecting individual responses to drugs, might help to optimize the initiation and maintenance dosage of tacrolimus to reach its target concentrations rapidly and to reduce its adverse reactions [7, 8]. At present, several single nucleotide polymorphisms (SNPs) have been studied in relation to the dosing of tacrolimus [9]. The predominant enzyme responsible for tacrolimus metabolism is CYP3A5 and the CYP3A5 A6986G polymorphism had a strong influence on tacrolimus elimination (^*∗*^3). The variant sequences A→G cause alternative splicing and the formation of a truncated CYP3A5 protein that is not functional. Consequently, individuals with homozygous allele CYP3A5^*∗*^3 (6986 GG) are often referred to as “CYP3A5 nonexpressers.” Patients with at least one CYP3A5^*∗*^1 wild-type allele (6986 GA/AA) are able to produce functional CYP3A5 enzymes and are known as “CYP3A5 expressers” [10]. The impact of CYP3A5 A6986G polymorphisms on tacrolimus dose requirements has been confirmed in liver, kidney, and lung transplantation studies [11–14]. Although the association between SNPs in CYP3A4, ABCB1, and other genes and tacrolimus elimination had been observed in numerous studies, research has been able to reliably show that only the CYP3A5 genotype can modify the pharmacokinetics of tacrolimus at present [15].

Cytokines are thought to be potent depressors of cytochrome P450 (CYP) enzymes and cause the depression of CYP-associated drug metabolism. The CYP gene expression and enzyme activities are downregulated during inflammation, infection, and transplantation rejection [16, 17]. The influence of interleukin-10 (IL-10) and interleukin-18 (IL-18) polymorphisms on tacrolimus pharmacokinetics had been described in liver and kidney transplantation. Li et al. [18, 19] found that the IL-10 G-1082A polymorphism in recipients was significantly associated with tacrolimus concentration/dose (*C/D*) ratios (IL-10-1082GG<GA<AA) within the first 3 weeks after liver transplantation; however, no significant effect of IL-10 G-1082A polymorphism was observed on population pharmacokinetic parameter of tacrolimus within 175 days after liver transplantation. Our previous study showed that the tacrolimus* C/D* ratios of the recipients with donors who were CYP3A5 nonexpressers and had low IL-10 production genotype (-819TT, -592 AA) were higher than those with donors who were CYP3A5 nonexpressers and had high IL-10 production genotype (-819CC or CT, -592CC or AC) in the first 2 weeks after liver transplantation [20]. Another study showed that the proportion of patients in the IL-10-819 TT group who achieved the target* C0* ranges was greater compared to the IL-10-819 CT/CC groups at week 3 after kidney transplantation [21].

The recipients with higher IL-18 serum levels had lower tacrolimus* C/D* ratios in liver transplantation [22]. Donor IL-18 rs5744247 polymorphism was an independent predictor of tacrolimus elimination in the first week after liver transplantation resulting from two independent cohorts [23]. IL-18 rs1946518 gene polymorphism was associated with log-transformed tacrolimus* C/D* ratios in Chinese kidney transplant recipients [24].

The target tacrolimus concentration, incidence rate of infection, and rejection in lung transplant recipients were higher than those in liver and kidney transplant recipients [4–6]. The expression of cytokines and tacrolimus metabolism varied in different kinds of transplantation. To date, the influence of IL-10 and IL-18 genetic polymorphisms on the pharmacokinetic parameters of tacrolimus remains unclear in lung transplantation.

In this study, we examined the IL-10 and IL-18 genotypes of lung transplant recipients to clarify the influence of these genetic variants on tacrolimus elimination after transplantation. We used the* C/D* ratio as an index of tacrolimus pharmacokinetics.

## 2. Patients and Methods

A total of 51 (7 female and 44 male) adult lung transplant recipients who received tacrolimus-based immunosuppressive regimens at Shanghai Pulmonary Hospital between July 2005 and July 2015 were included in this study. Transplant subtypes included 29 single right lung transplants, 16 single left lung transplants, and 6 bilateral lung transplants. All patients were Han Chinese. The mean age (±SD) was 54 ± 10 years (range: 28 to 75 years) and mean body weight was 58.5 ± 13.3 kg (range: 36 to 86 kg). Tacrolimus trough levels* C0* (ng/mL) were determined by Pro-TracIITM FK506 ELISA kit (DiaSorin, USA) with microparticle enzyme immunoassay (ELx 800NB analyzer, BioTek, USA). Dose-adjusted trough blood concentrations (*C/D* ratio, mg/kg body weight) in 4 weeks after transplantation were calculated. 68 normal (nondiseased) liver tissues were previously collected from Chinese donors in Shanghai First People's Hospital. The study was approved by the Ethics Committee of Shanghai Pulmonary Hospital and Shanghai First People's Hospital. Written informed consent was obtained from all patients in accordance with the Declaration of Helsinki and its amendments.

### 2.1. Genomic DNA Isolation and Genotyping

CYP3A5 rs776746, 2 SNPs of IL-18 (rs5744247 and rs1946518), and 3 SNPs of IL-10 (rs1800896, rs1800872, and rs3021097) were genotyped. Peripheral blood samples were collected in ethylenediaminetetraacetic acid (EDTA) tubes and preserved at −20°C before use. DNA was extracted from whole blood using a standard phenol-chloroform method. A multiplexed SNP MassEXTEND assay was designed by the Sequenom MassARRAY Assay Design 3.0 software (San Diego, CA, USA). The 6 SNPs were genotyped using the Sequenom MassARRAY RS1000 according to the standard protocol recommended by the manufacturer. Data were managed using the Sequenom Typer 4.0 software. Hardy-Weinberg equilibrium, allele frequency, and linkage disequilibrium were analyzed using SHEsis software (http://analysis.bio-x.cn/myAnalysis.php) [25].

### 2.2. Real-Time Reverse Transcriptase-PCR

Total RNA was extracted using RNeasy kit (Qiagen, Hilden, Germany) following the manufacturer's protocol. First-strand cDNA was synthesized using High Capacity cDNA Reverse Transcription Kit according to the manufacturer's instruction (Applied Biosystems, Carlsbad, CA, USA). Quantitative PCR was performed with SYBR Green PCR Master Mix (Applied Biosystems) and Mastercycler ep realplex (Eppendorf, Hamburg, Germany). IL-18 was amplified using the sense primer 5′-CCAAGGAAATCGGCCTCTAT-3′ and antisense primer 5′-TTGTTCTCACAGGAGAGAGTTGA-3′. *β*-Actin was amplified as endogenous control using the sense primer 5′-GGGTCAGAAGGATTCCTATG-3′ and the antisense primer 5′-GGTCTCAAACATGATCTGGG-3′ [26]. These reactions were repeated three times.

### 2.3. Statistical Analysis

Experimental data were analyzed using SPSS version 17.0 (SPSS, USA) and GraphPad Prism version 5.00 (http://www.graphpad.com/) software. The tacrolimus* C/D* ratios are expressed as median ± interquartile range. The difference in* C/D* ratios was compared using Mann–Whitney *U* test or Kruskal-Wallis test among groups. *t*-tests were performed to compare IL-18 mRNA in different genotypes. The statistical graphs were made by GraphPad Prism version 5.00 software (http://www.graphpad.com/). Two-tailed *P* values of less than 0.05 were considered statistically significant.

## 3. Results

### 3.1. Effect of Single SNP on Tacrolimus* C/D* Ratios

Allele and genotype frequency of the CYP3A5 rs776746, IL-18 rs5744247, IL-18 rs1946518, IL-10 rs1800896, IL-10 rs1800872, and IL-10 rs3021097 are shown in Table 1. All six SNPs were in accordance with Hardy-Weinberg equilibrium (*P* > 0.05). The effects of single donor SNP on tacrolimus* C/D* ratios are shown in Table 2. Tacrolimus* C/D* ratios of CYP3A5 rs776746 GG genotype carriers were 243.9 ± 244.2, 218.2 ± 211.9, 194.0 ± 212.2, and 156.8 ± 188.6 at week 1, week 2, week 3, and week 4, respectively. For AG and AA genotype carriers, the corresponding tacrolimus* C/D* ratios were 96.8 ± 145.6, 97.0 ± 143.0, 79.5 ± 130.6, and 80.6 ± 59.2. The differences were significant at week 1, week 2, week 3, and week 4 (*P* = 0.005, 0.008, 0.003, and <0.001, resp.). Tacrolimus* C/D* ratios of IL-18 rs5744247 GG+CG genotype carriers were 202.3 ± 241.1, 204.6 ± 181.0, 192.2 ± 186.7, and 115.7 ± 132.4 at week 1, week 2, week 3, and week 4, respectively. For CC genotype carriers, the corresponding tacrolimus* C/D* ratios were 92.4 ± 144.2, 79.2 ± 135.5, 74.8 ± 133.8, and 73.9 ± 110.3. The differences were significant at week 1, week 2, week 3, and week 4 (*P* = 0.002, 0.004, 0.005, and 0.026, resp.). Tacrolimus* C/D* ratios of IL-18 rs1946518 CC genotype carriers were 269.5 ± 239.0, 224.0 ± 192.4, 197.7 ± 226.3, and 151.0 ± 193.5 at week 1, week 2, week 3, and week 4, respectively. For CA genotype carriers, tacrolimus* C/D* ratios were 191.6 ± 192.2, 159.3 ± 194.6, 158.7 ± 158.6, and 108.1 ± 96.8 For AA genotype carriers, tacrolimus* C/D* ratios were 76.6 ± 108.8, 9.2 ± 86.3, 65.9 ± 84.4, and 73.9 ± 110.3 The differences were significant at week 1, week 2, and week 3 (*P* = 0.007, 0.018, and 0.019, resp.) and were nearly significant at week 4 (*P* = 0.079). Tacrolimus* C/D* ratios of IL-10 rs1800896 AA genotype carriers were 193.9 ± 224.79, 176.8 ± 205.14, 170.8 ± 186.69, and 104.8 ± 125.9 at week 1, week 2, week 3, and week 4, respectively. For AG genotype carriers, tacrolimus* C/D* ratios were 77.19 ± 66.4, 74.13 ± 32.24, 77.84 ± 41.57, and 85.55 ± 44.9 For AA genotype carriers, tacrolimus* C/D* ratios were 76.6 ± 108.8, 9.2 ± 86.3, 65.9 ± 84.4, and 73.9 ± 110.3 The differences were significant at week 1 and week 2 (*P* = 0.027 and 0.034, resp.). No significant difference of tacrolimus* C/D* ratios of IL-10 rs1800872 and rs3021097 was found in our data.

### 3.2. Associations between Combined IL-18 rs5744247 and rs1946518 Polymorphisms and Tacrolimus* C/D* Ratios

**Figure 1:**
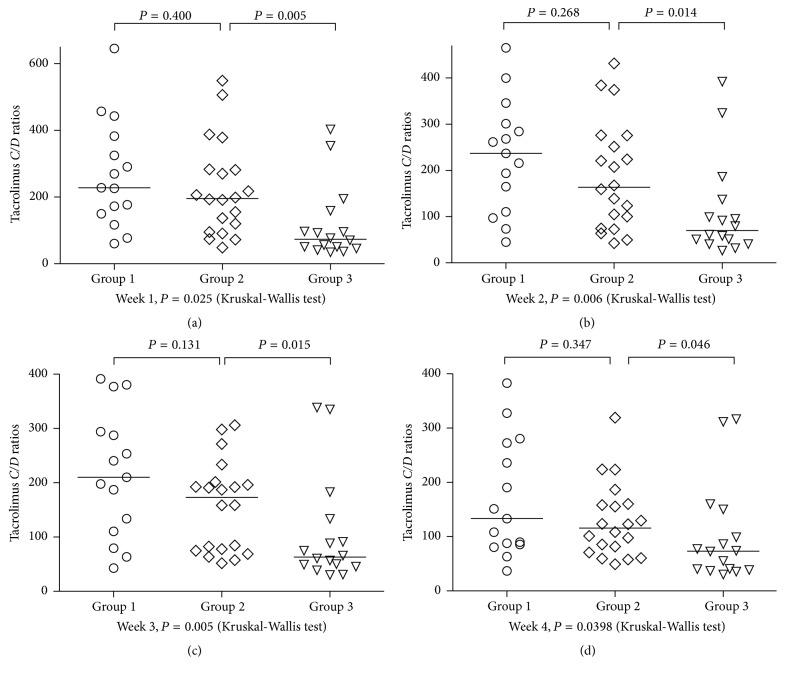
Combined effect of IL-18 rs5744247 and rs1946518 polymorphisms on tacrolimus concentration/dose (*C/D*) ratios in lung transplant patients at week 1 (a), week 2 (b), week 3 (c), and week 4 (d) following transplantation. IL-18 rs5744247 allele C and rs1946518 allele A were associated with fast tacrolimus metabolism. The lung transplant recipients were divided into 3 groups according to the numbers of these alleles: Group 1 (the number of alleles less than or equal to 1), Group 2 (the number of alleles equal to 2), and Group 3 (the number of alleles greater than or equal to 3). Patients who carried greater than or equal to 3 alleles associated with fast tacrolimus metabolism (Group 3) had lower tacrolimus* C/D* ratios than the other groups (Kruskal-Wallis test, *P* < 0.05).

IL-18 rs5744247 and rs1946518 were shown to be associated with tacrolimus elimination. Therefore, the two genotypes were further investigated in a combination analysis. IL-18 rs5744247 allele C and rs1946518 allele A were shown to be associated with fast tacrolimus metabolism as stated above. Patients in Group 1 carried less than or equal to 1 allele; patients in Group 2 carried 2 alleles; and patients in Group 3 carried greater than or equal to 3 alleles. Group 3 had lower tacrolimus* C/D* ratios than Group 1 and Group 2 (Kruskal-Wallis test, *P* < 0.05). No significant difference was found in tacrolimus* C/D* ratios of Group 1 and Group 2 (Figure 1).

### 3.3. Analysis of IL-18 Polymorphisms on Tacrolimus* C/D* Ratios in Lung Transplant Recipients Stratified by CYP3A5 Genotype

**Figure 2:**
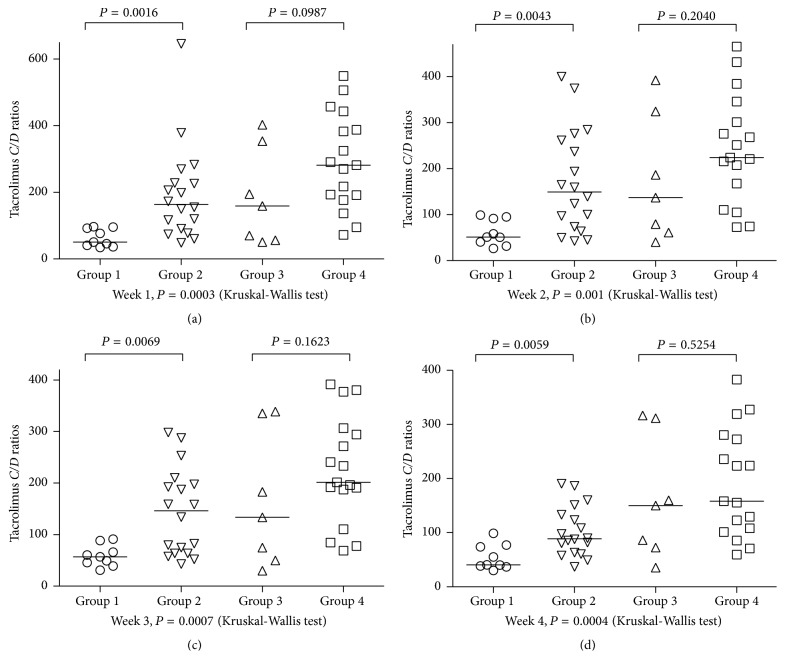
Combined effect of IL-18 rs5744247 and rs1946518 and CYP3A5 rs776746 polymorphisms on tacrolimus concentration/dose (*C/D*) ratios in lung transplant patients at week 1 (a), week 2 (b), week 3 (c), and week 4 (d) following transplantation. The lung transplant recipients were divided into 4 groups according to the number of IL-18 alleles associated with fast tacrolimus metabolism and CYP3A5 genotype: Group 1 (CYP3A5 expresser and the number of alleles greater than or equal to 3); Group 2 (CYP3A5 expressers and the number of alleles less than or equal to 2); Group 3 (CYP3A5 nonexpresser and the number of alleles greater than or equal to 3); Group 4 (CYP3A5 nonexpressers and the number of alleles less than or equal to 2). Among the patients with CYP3A5 expression genotype, the impact of IL-18 polymorphisms on tacrolimus* C/D* ratios was significant.

The lung transplant recipients were divided into 4 groups according to the number of IL-18 alleles associated with fast tacrolimus metabolism and CYP3A5 genotype. Patients in Group 1 were CYP3A5 expressers and carried greater than or equal to 3 alleles; patients in Group 2 were CYP3A5 expressers and carried less than or equal to 2 alleles; patients in Group 3 were CYP3A5 nonexpressers and carried greater than or equal to 3 alleles; patients in Group 4 were CYP3A5 nonexpressers and carried less than or equal to 2 alleles. Among CYP3A5 expresser recipients, the impact of IL-18 polymorphisms on tacrolimus* C/D* ratios was significant at week 1, week 2, week 3, and week 4 (*P* = 0.016, 0.043, 0.0069, and 0.0059, resp.). No significant influence of IL-18 polymorphisms on tacrolimus* C/D* ratios was observed in CYP3A5 nonexpresser recipients (Figure 2).

### 3.4. Influence of rs5744247 and rs1946518 on IL-18 mRNA in Liver Tissues

**Figure 3:**
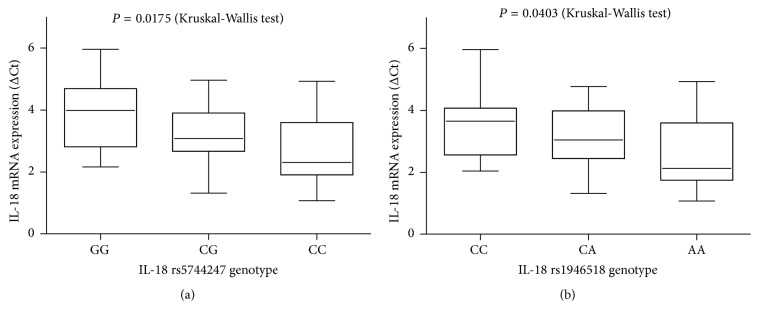
Analysis of IL-18 mRNA expressions in liver tissues with different IL-18 polymorphisms. IL-18 expression was determined by real-time PCR. IL-18 rs5744247 polymorphism was significantly associated with IL-18 mRNA expression (GG>CG>CC); IL-18 rs1946518 polymorphism was significantly associated with IL-18 mRNA expression (CC>CA>AA).

IL-18 rs5744247 polymorphism was significantly associated with IL-18 mRNA expression (GG>CG>CC, *P* = 0.175); IL-18 rs1946518 polymorphism was significantly associated with IL-18 mRNA expression (CC>CA>AA, *P* = 0.0403) (Figure 3).

## 4. Discussion

CYP3A5 rs776746 was a well-known biomarker for tacrolimus elimination [10–14]. In the present study, lung transplant recipients carrying CYP3A5 rs776746 AA/AG genotype had lower tacrolimus* C/D* ratios than those carrying CYP3A5 rs776746 GG genotype. Our data confirmed that CYP3A5 rs776746 polymorphism could be used to distinguish the tacrolimus elimination phenotype in lung transplantation. However, CYP3A5 rs776746 could not be applied to guide the individual dosage regimen of tacrolimus administered in routine transplant clinical practice for two reasons. First, CYP3A5 rs776746 can only classify patients into two categories with similar proportion in the Han Chinese. Second, the* C/D* ratios of the two categories had a large proportion of overlap [27, 28]. Further study is required to refine this process.

Rs1800896 (A-1082G), rs1800872 (A-592C), and rs3021097 (T-819C) were located in the promoter region of IL-10 and were the most IL-10 SNPs genotyped in previous studies [29, 30]. We found that the tacrolimus* C/D* ratios of IL-10 rs1800896 AA genotype carriers were significantly higher than of AG carriers in the first 2 weeks after transplantation. However, the minor allele frequency (MAF) of rs1800896 was 0.068 in the present study. The SNPs with low MAF (<5%) were usually rejected in association study [31]. As previously described, ethnicity can strongly influence the distribution of cytokine gene polymorphisms. In Caucasian patients, the IL-10 AA genotype at position -1082 occurred in 32.5% [32], while among Asian patients it occurred in 88.2% [33]. We thought the influence of rs1800896 on physiological and pathological phenomena in the Asian population had limited significance due to the low MAF of rs1800896.

In the present study, no significant difference of tacrolimus* C/D* ratios of IL-10 rs1800872 and rs3021097 was found in our data. This was inconsistent with the previous findings that IL-10 polymorphisms influenced tacrolimus pharmacokinetics in liver and kidney transplantation [18–21]. A meta-analysis including 595 rejection patients and 1239 stable graft patients suggested that the IL-10 gene polymorphisms (including rs1800896, rs1800872, and rs3021097) were not associated with an increased graft rejection risk in kidney transplantation [34]. Another meta-analysis suggested that IL-10 rs1800896 polymorphism may not be associated with acute rejection risk in liver transplantation recipients among Caucasians [35]. Also, there is no association between IL-10 polymorphisms (rs1800872 and rs3021097) and the incidence of acute rejection after lung transplantation [36]. The change of IL-10 mRNA level during rejection episode remained controversial [34]. There was clear evidence that the IL-10 rs1800872 and rs3021097 genotypes have an association with the decreased risk of infection in lung transplant recipients [36]. Incidence rate of infection in lung transplant recipients was higher than in liver and kidney transplant recipients. Therefore, we inferred that the protective effect of IL-10 polymorphisms on infection could confuse their influence on tacrolimus pharmacokinetics.

IL-18 rs5744247 and rs1946518 were two functional polymorphisms. IL-18 rs5744247 C>G variant reflects higher transcriptional activity and higher expression of IL-18 in LPS-stimulated monocytes and a higher serum IL-18 level [37]. IL-18 mRNA level significantly decreased gene expression in rs1946518 AA homozygotes (as compared with C allele carriers) in peripheral blood mononuclear cells [38]. Our data firstly showed that IL-18 rs5744247 and rs1946518 polymorphism was significantly associated with IL-18 mRNA expression in liver tissues. IL-18 rs5744247 C allele and rs1946518 A allele represented the low IL-18 mRNA expression allele. IL-18 is a member of the IL-1 family that has a homologous amino acid sequence to IL-1*β*. IL-1*β* has been shown to downregulate CYP3A protein by transcriptional and posttranscriptional mechanisms [39]. It had been demonstrated that IL-18 was a negative regulator of CYP3A5 mRNA expression in hepatocytes and could influence tacrolimus metabolism in liver transplant [23]. In the present study, we firstly proposed that IL-18 rs5744247 allele C and rs1946518 allele A were associated with fast tacrolimus metabolism and further combined analysis showed that the numbers of low IL-18 mRNA expression alleles had positive correlation with tacrolimus* C/D* ratios in lung transplant recipients.

In addition, our data showed that the influence of IL-18 polymorphisms on tacrolimus* C/D* ratios was observed in CYP3A5 expresser recipients, but not in CYP3A5 nonexpresser recipients.

In summary, our study suggested that* IL-18* rs5744247 and rs1946518 genotype contribute to differences in the* C/D* ratios of tacrolimus in lung transplantation patients. These findings may help to tailor tacrolimus administration in lung transplant recipients.

Our study has several limitations. First, the study population is confined to the Han Chinese nationality. The distribution of gene polymorphisms varies in different ethnicities. So, the conclusions of this study may not be applied to other ethnic populations. Second, this study is a single-center study and a small number of participants were involved. Further study with large sample sizes should been made to verify the association between cytokine gene polymorphisms and tacrolimus elimination.

## Figures and Tables

**Table 1:** Allele frequency of CYP3A5, IL-18, and IL-10 polymorphisms in lung transplant recipients (*n* = 51).

Gene	SNP	Genotype			Allele	
CYP3A5	rs776746	GG	AG	AA	G	A
		0.471 (24)	0.451 (23)	0.078 (4)	0.696 (71)	0.304 (31)

IL-18	rs5744247	GG	GC	CC	G	C
		0.118 (6)	0.510 (26)	0.373 (19)	0.373 (38)	0.627 (64)

IL-18	rs1946518	CC	CA	AA	C	A
		0.255 (13)	0.451 (23)	0.294 (15)	0.480 (49)	0.520 (53)

IL-10	rs1800896	AA	AG	GG	A	G
		0.863 (44)	0.137 (7)	0 (0)	0.931 (95)	0.069 (7)

IL-10	rs1800872	AA	AC	CC	A	C
		0.412 (21)	0.510 (26)	0.078 (4)	0.667 (68)	0.333 (34)

IL-10	rs3021097	TT	TC	CC	T	C
		0.431 (22)	0.490 (25)	0.078 (4)	0.676 (69)	0.324 (33)

**Table 2:** Comparison of tacrolimus concentration/dose ratios in different groups of CYP3A5, IL-18, and IL-10 polymorphisms at different times after drug initiation.

Gene	Locus	Genotype	Week 1		Week 2		Week 3		Week 4	
			*C/D* ratio	*P*	*C/D* ratio	*P*	*C/D* ratio	*P*	*C/D* ratio	*P*
CYP3A5	rs776746	GG	243.9 ± 244.2	0.005	218.2 ± 211.9	0.008	194.0 ± 212.2	0.003	156.8 ± 188.6	<0.001
		AG+AA	96.8 ± 145.6		97.0 ± 143.0		79.5 ± 130.6		80.6 ± 59.2	

IL-18	rs5744247	GG+CG	202.3 ± 241.1	0.002	204.6 ± 181.0	0.004	192.2 ± 186.7	0.005	115.7 ± 132.4	0.026
		CC	92.4 ± 144.2		79.2 ± 135.5		74.8 ± 133.8		73.9 ± 110.3	

IL-18	rs1946518	CC	269.5 ± 239.0	0.007	224.0 ± 192.4	0.018	197.7 ± 226.3	0.019	151.0 ± 193.5	0.079
		CA	191.6 ± 192.2		159.3 ± 194.6		158.7 ± 158.6		108.1 ± 96.8	
		AA	76.6 ± 108.8		79.2 ± 86.3		65.9 ± 84.4		73.9 ± 110.3	

IL-10	rs1800896	AA	193.9 ± 224.8	0.027	176.8 ± 205.1	0.034	170.8 ± 186.7	0.24	104.8 ± 125.9	0.24
		AG	77.2 ± 66.4		74.1 ± 32.2		77.8 ± 41.6		85.6 ± 44.9	

IL-10	rs1800872	AA	191.6 ± 232.3	0.985	167.5 ± 222.7	0.863	158.7 ± 228.8	0.909	98.8 ± 156.3	0.605
		CA+CC	165.8 ± 209.1		137.8 ± 171.2		133.8 ± 148.0		103.0 ± 86.3	

IL-10	rs3021097	CC+TC	159.2 ± 211.7	1.000	138.7 ± 179.2	0.909	133.9 ± 154.5	0.879	97.8 ± 86.8	0.634
		TT	184.3 ± 211.5		145.5 ± 217.2		134.6 ± 224.2		100.0 ± 142.5	

## Abbreviations

*IL-18*: Interleukin-18
*IL-10*: Interleukin-10
*CYP*: Cytochrome P450
SNP: Single nucleotide polymorphism
*C/D*: Serum concentration to dose ratio
EDTA: Ethylenediaminetetraacetic acid
PCR: Polymerase chain reaction.
